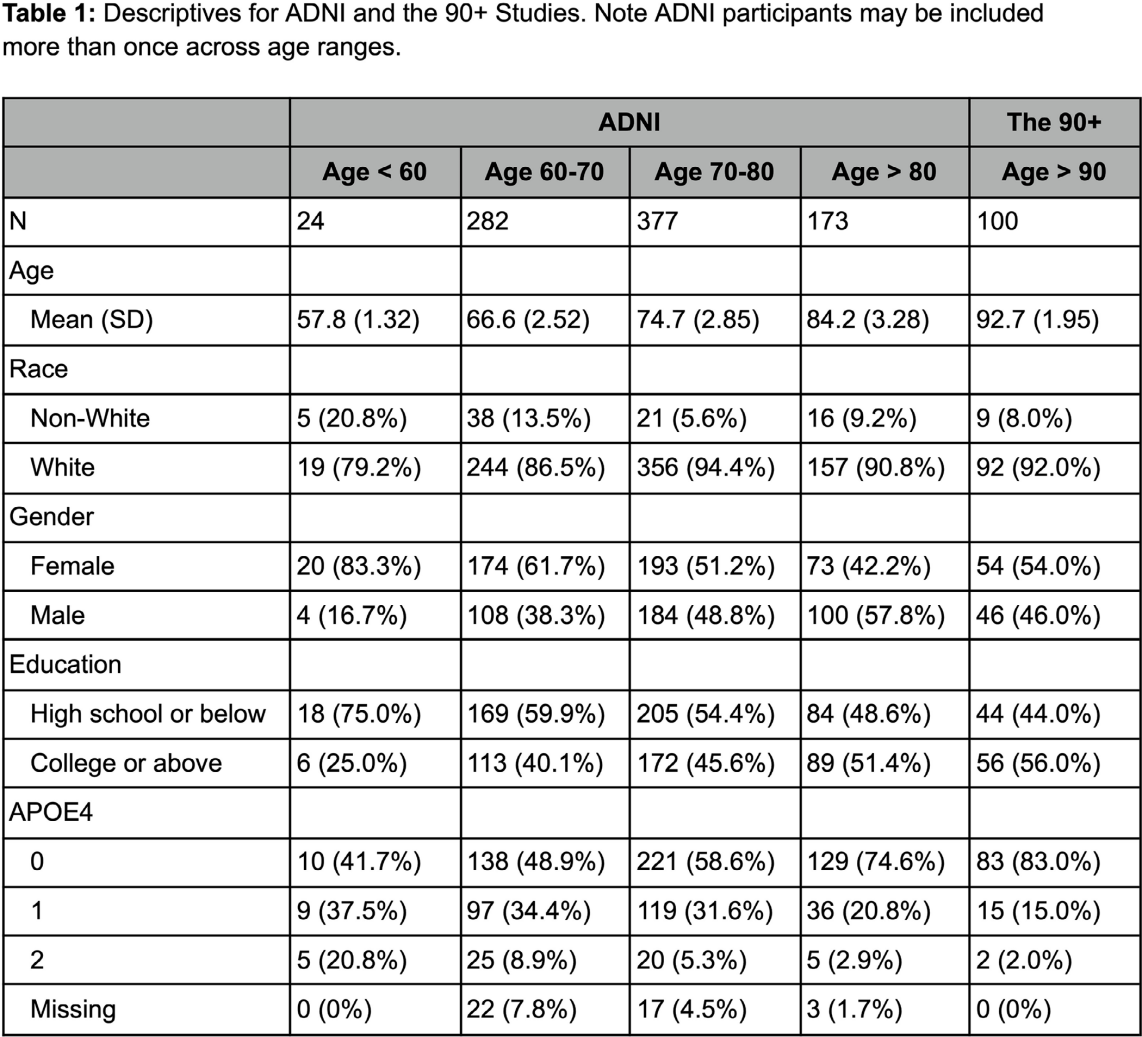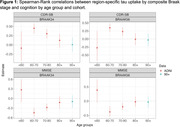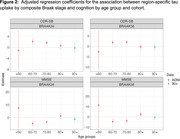# Association between Tau and Cognition as a Function of Age

**DOI:** 10.1002/alz.092900

**Published:** 2025-01-09

**Authors:** Sarah F Ackley, Jingxuan Wang, Charles Decarli, Evan Fletcher, Renaud La Joie, María M. M. Corrada, M. Maria Glymour

**Affiliations:** ^1^ Boston University, Boston, MA USA; ^2^ University of California, San Francisco, San Francisco, CA USA; ^3^ University of California, Davis, Davis, CA USA; ^4^ University of California, Irvine, Irvine, CA USA; ^5^ Boston University School of Public Health, Boston, MA USA

## Abstract

**Background:**

The relationship between tau pathology and cognition as a function of age and whether these relationships are consistent across samples is unknown. A growing number of studies now perform positron emission tomography with tau ligands (tau‐PET), making it possible for an improved understanding of the dynamics of tau and cognition in relation to other biomarkers.

**Method:**

We used data from the Alzheimer’s Disease Neuroimaging Initiative (ADNI) cohort (ages 55‐90) and The 90+ Study (Table 1) to cover a broad age range. We examined the association between region‐specific tau uptake by composite Braak stage and cognitive outcomes at the visit at which tau‐PET was performed (ADNI) or the closest visit (90+). We examined two cognitive outcomes: the mini mental status exam (MMSE) and clinical dementia rating sum of box scores. Spearman‐rank correlation coefficients between tau‐PET standardized uptake value ratios (SUVRs) with cerebellar cortex normalization and cognitive outcome are calculated for four distinct age ranges in ADNI: <60, 60‐70, 70‐80, and 80+ and among the 90+ study participants. Associations adjusted for age and age‐squared, race/ethnicity, number of APOE‐ε4 alleles, educational attainment, and gender are determined using bootstrapped linear regression.

**Result:**

The association between region‐specific tau uptake by composite Braak stage and cognition appears consistent for ages 60‐80 (Figures 1 and 2), with worse cognition with higher tau. Over age 80, tau does not appear to be associated with cognition. Based on point estimates, some associations appear to be reversed over age 80 in ADNI, which is not the case in the 90+ study, although these differences between cohorts are not statistically significant.

**Conclusion:**

Tau‐PET association varies with age, becoming less reliably associated with cognition over age 80. This is likely due to increased incidence of co‐occurring pathologies and selective survival. Associations may further depend on features of the source population.